# Breathing Well Across the Lifespan: Pulmonary Aging and Geroscience-Targeted Therapies

**DOI:** 10.1093/geroni/igaa057.2697

**Published:** 2020-12-16

**Authors:** Jason Sanders

## Abstract

Excellent pulmonary function is one of the strongest predictors of longevity across animal models and human populations. Unfortunately, none of the major age-associated pulmonary diseases – obstructive lung disease, pulmonary fibrosis, and increased susceptibility to pneumonia – have strongly effective disease modifying therapies. There is growing evidence that normal age-associated decline in pulmonary function and major age-associated pulmonary diseases are linked to the hallmarks of aging including senescence, nutrient signaling dysregulation, mitochondrial dysfunction, and telomere disorders. This presents opportunities for collaboration between gerontologists and pulmonologists to unravel age-associated developmental mechanisms and design novel treatments. In this symposium, leaders in pulmonary aging research will present novel data on links between aging and pulmonary health and geroscience-based interventions under study. Dr. Sanders will provide an overview of the scientific and clinical space and present epidemiologic associations between aging biomarkers, early pulmonary fibrosis, and mortality. Dr. Le Saux will discuss senescence and specifically how eicosanoid biology may explain organ-specific patterns of senescence-associated fibrosis. Dr. Thannickal will discuss age-associated perturbations in metabolism and mitochondrial function and targeting these pathways to improve lung function and treat pulmonary diseases. Dr. Newton will discuss mechanisms and clinical applications of telomere biology to pulmonary aging. Symposium attendees will (1) be poised to generate collaborations between gerontologists and pulmonologists to address existing knowledge gaps in mechanisms of pulmonary aging, and (2) develop a better understanding of translational opportunities to design geroscience-based diagnostics and therapeutics to improve pulmonary health with aging.

